# Jaw claudication and jaw stiffness in giant cell arteritis: secondary analysis of a qualitative research dataset

**DOI:** 10.1093/rap/rkad082

**Published:** 2023-10-24

**Authors:** Joyce Lim, Emma Dures, Lawrence F Bailey, Celia Almeida, Carlee Ruediger, Catherine L Hill, Joanna C Robson, Sarah L Mackie

**Affiliations:** Department of Rheumatology, Leeds Teaching Hospitals NHS Trust, Leeds, UK; School of Health and Social Wellbeing, University of the West of England, Bristol, UK; Academic Rheumatology, Bristol Royal infirmary, Bristol, UK; Patient and Public Involvement Group, Leeds Biomedical Research Centre, Leeds, UK; School of Health and Social Wellbeing, University of the West of England, Bristol, UK; Rheumatology Unit, The Queen Elizabeth Hospital, Woodville, South Australia, Australia; and the University of Adelaide, Adelaide, South Australia, Australia; Rheumatology Unit, The Queen Elizabeth Hospital, Woodville, South Australia, Australia; and the University of Adelaide, Adelaide, South Australia, Australia; School of Health and Social Wellbeing, University of the West of England, Bristol, UK; Academic Rheumatology, Bristol Royal infirmary, Bristol, UK; Leeds Biomedical Research Centre, Leeds Teaching Hospitals NHS Trust, Leeds, UK; Leeds Institute of Rheumatic and Musculoskeletal Medicine, University of Leeds, Leeds, UK

**Keywords:** giant cell arteritis, temporal arteritis, jaw claudication, symptoms

## Abstract

**Objective:**

Jaw symptoms can be a vital clue to the diagnosis of GCA. Guidelines recommend more intensive treatment if jaw claudication is present. We sought to explore how patients with GCA described their jaw symptoms.

**Methods:**

We carried out a secondary, qualitative analysis of interview data from 36 participants from the UK (*n* = 25) and Australia (*n* = 11), originally collected in order to develop a patient-reported outcome measure for GCA. In all cases, GCA had been confirmed by biopsy/imaging. Interview transcripts were organized within QSR NVivo 12 software and analysed using template analysis. Themes were refined through discussion among the research team, including a patient partner.

**Results:**

Twenty of 36 participants reported jaw symptoms associated with GCA. The median age of these 20 participants was 76.5 years; 60% were female. Five themes were identified: physical sensations; impact on function; impact on diet; symptom response with CSs; and attribution to other causes. Physical sensations included ache, cramp, stiffness and ‘lockjaw’. Functional impacts included difficulty in eating/chewing, cleaning teeth, speaking or opening the mouth. Dietary impacts included switching to softer food. Response to CSs was not always immediate. Jaw symptoms were initially mis-attributed by some participants to arthritis, age or viral illnesses; or by health-care professionals to a dental cavity, ear infection or teeth-grinding.

**Conclusion:**

Jaw symptoms in GCA are diverse and can lead to diagnostic confusion with primary temporomandibular joint disorder, potentially contributing to delay in GCA diagnosis. Further research is needed to determine the relationship of jaw stiffness to jaw claudication.

Key messages

- Jaw symptoms in GCA include stiffness, in addition to claudication.
- Both stiffness and claudication can affect jaw function, leading to dietary modification.
- Jaw symptoms of GCA may be mis-attributed to other causes, causing diagnostic delay.

## Introduction

GCA is the commonest primary systemic vasculitis. Early diagnosis and prompt treatment with high-dose glucocorticoid are crucial to avert visual loss. Jaw claudication in GCA was originally described by Horton as pain that ‘occurred only with chewing and promptly disappeared with rest’ [1]. It has been formally defined as ‘development or worsening of fatigue in the muscles of mastication while eating’ [2] and is understood as a manifestation of ischaemia to these muscles. In a recent meta-analysis, jaw claudication was the GCA feature with highest diagnostic odds ratio [3]. Furthermore, patients presenting with jaw claudication are more likely to have visual loss at presentation [4].

EULAR recommends that return of active GCA accompanied by jaw claudication constitutes ‘major relapse’ and requires treatment as for new-onset disease, whereas ‘minor relapses’ can be treated by returning to the last effective glucocorticoid dose [5]. For disease relapse with symptoms of cranial ischaemia, the ACR guidelines recommend adding a non-glucocorticoid immunosuppressant and increasing the glucocorticoid dose [6]. Jaw claudication, by definition a cranial ischaemic symptom, thus has major implications for both diagnosis and treatment of GCA.

In 1962, Horton wrote: ‘I finally realized, during the years 1942–44, that the pain of chewing referred to by patients as difficulty in chewing or “lockjaw” is an exercise phenomena and represents intermittent claudication of the jaw’ [7]. Reminiscent of Horton’s words, a recent international qualitative study [8] to develop a patient-reported outcome measure reported that patients with GCA described jaw stiffness in addition to jaw pain. In this study, we conducted a secondary analysis of this qualitative dataset with the aim of exploring the range of patient-reported jaw symptoms in GCA.

## Methods

### Design

We conducted a secondary analysis of qualitative data from interviews conducted for a study to develop a patient-reported outcome measure for GCA [8]. The primary analysis found that participants described a variety of jaw symptoms; this secondary analysis was therefore conducted to explore this aspect further. The original study obtained ethical approval in the UK (South Central—Oxford B Research Ethics Committee; REC reference: 16/SC/0697, IRAS project ID: 217748) and Australia (Central Adelaide Local Health Network; HREC reference: HREC/17/TQEH/275 and CALHN reference: Q20170906). All patients provided written informed consent.

### Data collection and participants

In the original study, participants were recruited using purposive sampling from two rheumatology clinics and one ophthalmology clinic in the UK (*n* = 25) and Australia (*n* = 11). All participants had a definite diagnosis of GCA confirmed by at least one diagnostic test (temporal artery biopsy, temporal artery US, CT angiogram or PET scan). Interviews were recorded, transcribed and anonymized. In the present study, transcripts were organized within one QSR NVivo 12 database.

### Analysis

*A priori* themes, namely description of jaw symptoms, impact on daily activities and impact on diet, were determined from initial discussion among the researchers (J.L., E.D., J.R. and S.L.M.) to facilitate the initial coding process. These were selected based on clinical significance and relevance to the research question. J.L. read all the anonymized transcripts and coded segments of text related to the research question and topic. A subset of transcripts was initially coded. Codes were then grouped into themes and sub-themes, and the whole dataset of interviews was re-reviewed to ensure completion of data.

J.L. discussed the analysis with J.R., E.D., S.L.M. and a patient partner (L.F.B.) to ensure that different viewpoints were considered. Themes were redefined and revised following these discussions.

## Results

Of the 36 participants with GCA, 20 (58%) reported jaw symptoms. Of these, the median age was 76.5 years; 60% were female. Specific demographics have been described previously [8].

### Physical sensations

Physical sensations reported were primarily in the jaw and, to a lesser extent, in the ear and face.

Participants commonly reported jaw pain/ache/cramp that occurred when eating and resolved with rest.

To eat a meal, I’d have to keep stopping, because my jaw ached…and I’d have to stop, rest and then eat a bit more and stop and rest and carry on like that. (Female, 83 years old, UK)

I could only do a few attempts at chewing something and swallowing it. Internally, deep inside, it became sore and a sort of cramp… (Male, 79 years old, Australia)

Several participants described jaw stiffness or ‘lockjaw’.

…because my first symptom of all was the jaws, very stiff jaws, very painful to eat… (Female, 72 years old, UK)

…and a very stiff jaw, which affected my eating. (Male, 81 years old, UK)

I get a lock in the jaw up here sometimes, which is the same side as what I had, you know. (Male, 72 years old, UK)

Jaw symptoms were sometimes associated with other non-headache symptoms, including pain or altered sensation in the ear (or around the back of the ear), face, teeth and tongue. Some participants reported ‘swelling’ of the mouth or tongue.

### Impact on function

Jaw symptoms impacted oral functions including chewing, cleaning the teeth, opening the mouth and speaking.

Yes, the jaw ache, that was what worried me then, and then I could hardly bite an apple… (Female, 78 years old, UK)

…I got to the stage when occasionally I couldn’t open my mouth to, er, clean with a small electric toothbrush, I guess… (Female, 78 years old, UK)

When pain was severe, it could impact on sleep.

…it was difficult to sleep. Oh, everything, and it was absolutely excruciating, I told him what such pain I’m in, I said, ‘I can hardly speak, I can hardly open my mouth’… (Female, 64 years old, UK)

### Impact on diet

Dietary modifications reported by participants included a shift to a soft or liquid diet. This was not only attributable to classical jaw claudication symptoms but also to their difficulty in opening the mouth wide enough to accommodate a sandwich or even a large spoon.

All me jaws was hurting, oh that was painful.… So, I was living on soups and tins of rice. (Female, 79 years old, UK)

…and I was having to use a teaspoon for cereal… (Female, 78 years old, UK)

Some participants reported eating less and losing weight, whereas others reported eating more slowly but not necessarily eating less.

### Response to glucocorticoid therapy

Some participants reported an immediate response of jaw symptoms to glucocorticoid therapy. For others, the symptoms were much slower to resolve.

### Attribution to other causes

Participants attempted to make sense of their symptoms in various ways, often attributing their jaw symptoms to arthritis, old age or intercurrent infection.

I probably put up with it for a long time, because of other aches and pains, and I’ve got arthritis in my jaw now, sort of thing. (Female, 66 years old, UK)

I just—I think it’s something to do with my age, you know, you don’t go to the doctor. (Female, 71 years old, Australia)

I just put it down to, you know, being in the cabin at the, erm, aircraft, how you often get these viruses and things, erm, and so I didn’t take a lot of notice of it. (Female, 73 years old, UK)

One participant with PMR initially surmised that their jaw symptoms were attributable to their glucocorticoid therapy:

Pain in the teeth and jaw, terrible pain; I woke up at about 3 o’clock every morning thinking these are probably the side effects of the medication I’m taking for the polymyalgia—I hadn’t been warned at that stage that I could have got giant cell. (Female, 71 years old, Australia)

On seeking advice from health-care professionals, jaw symptoms were not always recognized as GCA immediately.

Because I had an actual appointment within a few days. And the dentist said, ‘There’s nothing really wrong, unless you may have, um, a cavity or something’. And she said, ‘Gargle with salt water.’. (Female, 64 years old, UK)

‘No, it’s fine’, he [eye doctor] said, went like this, whatever, ‘Grinding your teeth’, he said, ‘That’s the problem, nothing’s wrong, off you go’… (Female, 64 years old, UK)

…he [general practitioner] thought the antibiotics would have cured, ’cos he thought I had an infection. (Female, 64 years old, UK)

They said, ‘Well, perhaps it’s middle ear infection’. (Female, 67 years old, UK)

Table 1 provides further illustrative quotations under these themes.

**Table 1.** Overview of themes and sub-themes with illustrative quotes

Themes	Selected quotations
**1. Physical sensations**	
Jaw pain	There was a little bit of pain around I suppose the jaw line where the teeth are. (Male, 85 years old, Australia) I’m halfway through the panini and I realize I can’t chew because my jaw has started to ache… (Female, 75 years old, UK) I had jaw pain … I seemed to have to take erm, aspirin or something to erm, before I had my breakfast so that I could open my mouth properly … to have my breakfast. (Female, 86 years old, UK) I had a sandwich and I thought ‘Cor’, you know, me jaw joint um, hurt, was quite painful. (Male, 72 years old, UK) To eat a meal, I’d have to keep stopping, because my jaw ached … and I’d have to stop, rest and then eat a bit more and stop and rest and carry on like that. (Female, 83 years old, UK)
Cramp	I could only do a few attempts at chewing something and swallowing it. Internally, deep inside, it became sore and a sort of cramp… (Male, 79 years old, Australia)
Jaw stiffness	…a very stiff jaw which affected my eating. (Male, 81 years old, UK) …because my first symptom of all was the jaws, very stiff jaws, very painful to eat… (Female, 72 years old, UK) …then there was a bit of stiffness in my jaw erm, my tongue felt quite strange … the stiffness in the jaw was the main thing. Not really headache-y, erm a tension sort of in the skull and I felt as though my skull was actually like erm an ice bucket because when I went to bed at night I felt as though I needed, well I wrapped it in like towels. (Female, 73 years old, UK)
Lockjaw	Then it would go down to the eyes and it would give you lockjaw. (Male, 80 years old, Australia) I get a lock in the jaw up here sometimes which is the same side as what I had (the headache), you know. (Male, 72 years old, UK) ‘I can hardly speak, I can hardly open my mouth’, and I, it was as if, you went like this with your jaw and I thought ‘Is it lockjaw?’. (Female, 64 years old, UK)
Ear pain	The back of, of the sort of, the back of my head, er sort of just alongside the ear, the sort of bone there, that, that quite often, I get an ache there and that sometimes goes up into my head… (Female, 69 years old, UK) I felt I had a pain in my ear, but not in the ear … around the ear… (Female, 78 years old, UK) But it was much more sort of just generally around the head and behind where the ear is, I think it was. (Female, 67 years old, UK) It’s sort of a pain behind the ear. (Female, 74 years old, UK)
Face pain	A few weeks later, I developed a very intense pain on the left-hand side of the face. (Male, 73 years old, UK) I woke up on a Monday morning with my face just aching. All the bones were just aching in the face. (Female, 83 years old, Australia)
Teeth pain	Pain in the teeth and jaw, terrible pain. (Female, 71 years old, Australia)
Tongue ache, felt too big	…me jaw ached and me tongue ached. (Male, 72 years old, UK) …and the tongue felt as though it was too big for the mouth, it was a funny thing to sort of say … this big tongue in the mouth business as though it's swollen. (Female, 73 years old, UK)
Swelling	’Cos I couldn’t open my mouth um and all this was all swollen, like great, huge, um and I was on um two lots of strong antibiotics. (Female, 64 years old, UK)
**2. Impact on function**	
Jaw/mouth opening	…and I could open my mouth normally… (Female, 78 years old, UK) He got to a point where he couldn’t even take a bit of soup because it was hard to open his mouth (Male, 80 years old, Australia) That was the very … that was my very first symptom, because I was in bed and I thought, ‘I can’t really open my mouth very wide’. That was my very first symptom. (Female, 72 years old, UK) I couldn’t open my mouth to eat properly it was, I could only open it very little. (Female, 74 years old, UK)
Moving face	Although you want to, you couldn’t move the face, facial muscles either, ’cos you couldn’t smile, your face is like, yeah like, like a mask really. (Female, 64, UK)
Chewing/Eating	I couldn’t put my teeth together so I couldn’t chew - my mouth was not fully open. (Female, 71 years old, Australia) When you chewed you chew half a dozen times. Then it would ache then you would stop, then you would continue on and so on. (Male, 85 years old, Australia) And then, I started getting, I couldn’t eat. All me jaws was hurting, oh that was painful… So, I was living on soups and tins of rice. (Female, 79 years old, UK) Yes, the jaw ache that was what worried me then, and then I could hardly bite an apple… (Female, 78 years old, UK)
Cleaning teeth	…I got to the stage when occasionally I couldn’t open my mouth to, er, clean with a small electric toothbrush, I guess… (Female, 78 years old, UK)
Speaking	But it was … the first signal was chewing, I could not chew and I couldn’t use my jaw, it hurt to talk, it hurt to move it… (Female, 75 years old, UK) …I could hardly speak, it was so embarrassing, to try and, it’s as if someone’s clamped the jaw shut and you. (Female, 64 years old, UK)
Sleeping	…it was difficult to sleep. Oh, everything, and it was absolutely excruciating, I told him what such pain I’m in, I said, ‘I can hardly speak, I can hardly open my mouth’, and I, it was as if you went like this with your jaw, and I thought, ‘Is it lockjaw?’ (Female, 64 years old, UK)
**3. Impact on diet**	
Change in food consistency	It was really, really difficult to eat. And in the end, I had to have, all I could have was soups, liquid foods. (Female, 64 years old, UK)
Change in cutlery	…and I was having to use a teaspoon for cereal… (Female, 78 years old, UK)
Eating less	…I was not eating so much, so I was losing weight… (Female, 72 years old, UK)
Eating slower	When my jaw wasn’t, erm, I’d be slower eating but I don’t think I ate less. (Female, 74 years old, UK)
**4. Symptom response with steroids**	
Fast response	The jaw pain… Yes, it was very quickly… (Female, 66 years old, UK) Er, it alleviated it straightaway, the prednisolone did. (Male, 80 years old, UK)
Slower response	But the jaw took quite some time, that seemed to be the longest time. (Female, 74 years old, UK)
**5. Attribution to other causes**	
Health-care professionals: dental cavity, infection, teeth grinding	Because I had an actual appointment within a few days. And the dentist said, ‘There’s nothing really wrong, unless you may have um a cavity or something'. And she said, ‘Gargle with salt water’. (Female, 64 years old, UK) …he [general practitioner] thought the antibiotics would have cured, ’cos he thought I had an infection. (Female, 64 years old, UK) They said, ‘Well, perhaps it’s middle ear infection’. (Female, 67 years old, UK) ‘No, it’s fine’, he (eye doctor) said, went like this, whatever, ‘Grinding your teeth’, he said, ‘That’s the problem, nothing’s wrong, off you go.’… (Female, 64 years old, UK)
Patient: arthritis, age, viral illness	I probably put up with it for a long time, because of other aches and pains, and I’ve got arthritis in my jaw now sort of thing. (Female, 66 years old, UK) …but I mean they were that sort of, er, very very slight twinge that you would never have sort of gone to the doctors with that. (Male, 70 years old, UK) I just—I think it’s something to do with my age, you know, you don’t go to the doctor. (Female, 71 years old, Australia) I just put it down to, you know, being in the cabin at the erm aircraft, how you often get these viruses and things erm and so I didn’t take a lot of notice of it. (Female, 73 years old, UK)

## Discussion

We conducted a secondary analysis of interview data and found a wide range of jaw symptoms in GCA. For some participants, ‘stiffness’ of the jaw affected functions related to mouth opening, including talking, teeth cleaning or accommodating cutlery. Other authors have previously reported that reduction in jaw opening (trismus) is ‘an overlooked feature of GCA’ [9, 10]. This could be detected upon clinical examination of the jaw or might also be described by patients as difficulty in mouth or jaw opening [10]. For some participants, difficulty in opening the mouth had an even greater functional impact than difficulty in exerting force during chewing; some participants compensated for this by eating more slowly, whereas others shifted to a soft or liquid diet. Some reported weight loss. Misattribution of jaw symptoms not typical for ‘textbook’ jaw claudication might have contributed to diagnostic delay in some patients. Many of the accompanying symptoms of the tongue or ear would have been compatible with GCA but might also have served as ‘red herrings’ until the GCA diagnosis was suspected.

**Figure 1.**
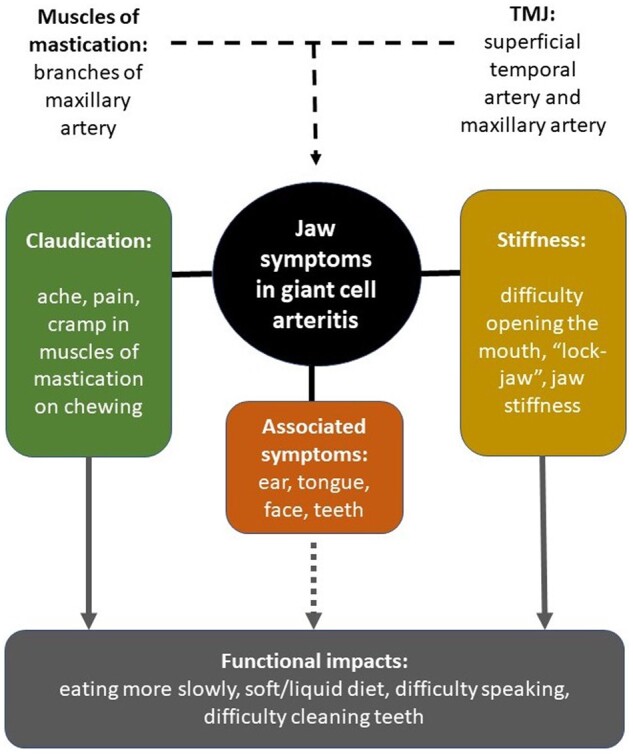
A proposed typology of jaw symptoms in giant cell arteritis

The external carotid artery has two terminal branches: the superficial temporal artery and the maxillary artery. Branches of the second part of the maxillary artery (deep temporal artery, masseteric artery and pterygoid branch) supply the muscles of mastication (temporalis, masseter and medial pterygoid), which are responsible for biting down with force, likely to be involved in the ‘textbook’ jaw claudication symptoms commonly reported by patients with GCA. The pterygoid branch of the maxillary artery also supplies the lateral pterygoid muscle, assists in opening the mouth and is necessary for the precise positioning of the mandible required for speaking [11] (Fig. 1).

Mouth opening can usually be accomplished by gravity alone; therefore, ‘stiffness’ or difficulty in opening the mouth suggests that the temporomandibular joint itself might sometimes be affected in GCA. In line with Horton’s original suggestion, this too could be an ischaemic phenomenon. The temporomandibular joint itself is supplied by a rich arterial plexus, mostly from the superficial temporal artery, with some contribution from the maxillary artery [12].

Jaw claudication has a well-established association with visual loss, but the same might be true of jaw stiffness: reduced jaw opening (trismus), present in 7% of GCA patients, is associated with visual manifestations in GCA [10]. It is currently unclear whether jaw stiffness should be considered an ischaemic manifestation with the same diagnostic and treatment implications as jaw claudication in GCA.

A limitation of our study is that it was a secondary analysis; the original data collection was not designed to answer our specific question, and clarifying questions about jaw symptoms were not part of the original interview protocol. Therefore, although all participants had GCA confirmed by biopsy or imaging, we did not ask about a history of prior temporomandibular joint disorder. However, the symptoms were all glucocorticoid responsive. Lastly, participants came from the South of England and Australia; language used for jaw symptoms might differ elsewhere.

This is the first study to describe jaw symptoms in GCA from the perspective of patients. Participants recruited had different demographics, educational levels and disease characteristics, ensuring that perspectives from various backgrounds were considered [13]. People with GCA reported not only jaw claudication but also jaw stiffness and difficulties with mouth opening that affected many important everyday functions. In the context of previous literature, it appears likely that these are part of the spectrum of GCA symptoms of which clinicians, including dentists and ophthalmologists, should be aware. Confirmation of this suggestion and investigation of the implications for disease stratification and treatment require further research.

## Data Availability

Data are available upon request, please contact Dr Joanna Robson.
